# Supplementary material for: Are carbon emissions trading and green financial instruments synergistic? -Comprehensive quantitative research based on content analysis
Source: PLoS One. 2024 Mar 7;19(3):e0298601. doi: 10.1371/journal.pone.0298601 (PMC10919601; doi:10.1371/journal.pone.0298601)
Supplement: S1 Data — (ZIP) [file pone.0298601.s001.zip › Data/地级市Z_F工作报告.zip!/╔┬╬≈/╙▄┴╓╩╨/└┤╫╘╣1⁄2╓┌║┼í╛┬φ┐╦╩2╛▌═°í┐_2017.docx]

各位代表：

现在，我代表市人民政府向大会报告工作，请予审议，并请市政协委员和其他列席人员提出意见。

一、2016年工作回顾

2016年，是我市直面挑战、砥砺奋进、加快追赶超越的一年。一年来，面对实体经济运行困难、市场信心普遍不足、财政收支矛盾凸显、各类风险增多交织等不利局面，全市上下认真学习习近平总书记系列重要讲话精神，深入落实中省和市委重大决策部署，统筹推进稳增长、促转型、防风险各项工作，经济发展企稳向好，人民生活持续改善，实现了“十三五”的良好开局。全年实现地区生产总值2773亿元、增长6.5%，其中一产和三产增速分别达4.8%和11.1%，均居全省第一；完成全社会固定资产投资1467.5亿元、增长6%，扭转了连续三年下滑局面；完成地方财政收入232.7亿元；实现社会消费品零售总额420亿元、增长6%；城乡居民人均收入达29781元和10582元，分别增长7.3%和8%。圆满完成四届人大一次会议确定的目标任务，在全省2016年度目标责任考核中获得优秀等次。

一年来，我们主要做了以下工作：

（一）全力以赴稳增长。出台促进经济平稳增长若干意见、加强中省驻榆企业调度等一系列稳实体政策措施，设立1亿元工业稳增长专项资金，规上工业企业利润总额增长67.8%，工业增加值位居全省第一。出台扩大有效投资考核办法等针对性举措，组织实施重点项目90个，实质性开工PPP项目14个，民间投资降幅同比收窄6.8个百分点，新开工项目增长42.2%。着力提升消费拉动作用，出台加快发展服务贸易的实施意见，启动榆林消费升级行动计划，积极拓展健康、养老、家政等消费新领域，服务业增加值比重提高到33.5%。加快发展文化旅游产业，新增3A级景区5家，佳县成功创建省级旅游示范县，举办“清爽榆林”“陕北榆林过大年”等系列活动，接待游客和旅游收入分别增长24%和33%。持续加强外向型经济建设，实现海关通关一体化，新增出口国家和地区25个，货物进出口总额增速跃居全省第一，十一届“煤博会”获2016年全国十大特色会展优秀奖。

（二）聚焦产业促转型。深入实施创新驱动战略，新建煤炭清洁利用、新材料、旱区农业节水3个市级工程技术中心，设立1.5亿元的科技成果转化引导基金，大型煤炭分质清洁高效转化关键技术研发与应用示范项目被列为全省首个重点产业创新链，“星创天地”工作走在全国前列，科技型企业产值增长14%。全力推进能化基地高端化，启动工业转型升级三年行动计划，新批煤电装机398万千瓦，新增煤化工产能200万吨，榆林成为全国煤制烯烃产能第一大市；神木锦丰源、陕煤化乾元公司等粉煤热解技术取得重大突破，兰炭在国家层面被列为洁净煤，成为京津冀鲁地区替换烟煤的主力军。持续壮大现代农业，新增优质山地苹果8万亩，新认定市级农业园区16个，新申报无公害农产品产地50个、产品67个，粮食总产量突破160万吨。积极发展新产业新业态，非能源工业投资增长36.7%、产值增长15.5%，神木银丰陶瓷、融和1，4-丁二醇、定边智诚润滑油等一批转型项目建成投产，新增新能源装机290万千瓦，战略性新兴产业增加值增长11.1%。神南“煤亮子”平台上线运营，新注册电商企业436户，久旭快递物流园、阿里巴巴农村淘宝米脂服务中心建成投运，“互联网+”助推传统服务业加速转型。

（三）突出重点抓改革。供给侧结构性改革成效明显，全面完成省上下达的去产能任务，4400万吨煤炭产能减量置换方案获批；用活棚改货币化安置、移民搬迁等去库存政策，全市去库存周期由年初的106个月下降到23个月，中心城区降至20个月；全面落实降本增效政策措施，社保缴费阶段性调减2.53亿元，直供电交易40.5亿度、降低用电成本1.9亿元，兰炭尾气发电实现全额上网。“放管服”改革深入推进，承接中省取消下放的行政审批事项23项，行政职权精简16.6%；“五证合一、一照一码”稳步推开，新注册市场主体增长8.9%；“多规合一”试点成果获省政府批复，投资项目立项阶段并联审批全面启动；在全省率先建成不动产统一登记信息平台，实现“发新停旧”。事业单位改革稳步推进，25所中职学校整合到17所，榆林传媒中心挂牌。农村综合改革有序推进，农村土地确权登记颁证率达到92%，启动县级农村产权交易市场建设，建成榆林市土地承包经营权登记流转服务平台，推开集体产权制度改革试点。

（四）统筹城乡促协调。扎实推进中心城市建设，横山撤县设区正式挂牌，中心城区发展进入新的历史阶段；实施55个市政项目，完成投资25亿元，长城南路延伸段等13个路桥项目建成通车，13个公共停车场、161个公共自行车站、环城北路供水加压站等一批公益项目建成投运，新增公交线路4条；滨水西岸等7个棚改项目完成融资42.4亿元；新增集中供热230万平米、绿地28.1万平米，顺利通过省级生态园林城市验收。加快新型城镇化进程，启动长城沿线城镇带、无定河全线综合整治规划编制工作，大柳塔镇级小城市综合改革试验区正式揭牌，榆阳区金鸡滩等9个镇被列为国家级重点镇。全面启动美丽宜居乡村创建，绥德虎墕等13个村入选第四批中国传统村落，佳县赤牛坬等4个村被评为国家级美丽宜居示范村。强化振南工作，落实市级专项资金、北部县区对口帮扶资金3.06亿元，组织实施一批基础设施、公共服务和重大产业项目，南部县发展内生动力不断增强。

（五）集中力量补短板。基础设施建设取得突破，靖神铁路、神瓦铁路、榆林榆阳机场扩建等一批重大交通项目获批，延安—榆林高铁完成项目工可研，完成县乡公路改造216公里，新建通村公路1430公里，全省首条以PPP模式建设的绥延高速开工，县县通高速的目标即将实现；榆横—潍坊、陕北—关中二回2条电力外送通道加快建设，陕北—湖北特高压输变电工程列入国家“十三五”开工项目；定边扬黄改建提升、清涧黄河引水等水源工程有序推进。生态环境持续优化，完成营造林73.3万亩，榆阳区黄家圪公式、神东大柳塔矿区建成国家级水土保持示范园；严肃查处环保违法违规企业129家，关闭、取缔138家，清理整顿2280个环保违规项目，中心城区二级以上天数达到296天，空气质量居全省前三。金融生态不断改善，加大失信行为惩戒力度，发布4批诚信“红黑名单”；加大非法集资案件追缴处置兑付力度，案件受理数量下降65.7%，涉案群众下降59.9%；加强政银企对接，累计发放“助保贷”9.9亿元，发行各类债券37.8亿元，一批重点企业的存量债务得到有效化解，金融机构不良贷款占全省比重下降8.4个百分点。

（六）凝心聚力惠民生。民生支出383.3亿元，超额完成“两个80%”硬指标。强力推进脱贫攻坚，全年实现22.84万人脱贫、519个贫困村退出、定边县“摘帽”，榆阳区“医疗救助减贫脱贫”成为全国社会救助领域五佳成果之一。巩固十五年免费教育，完成学校标准化建设和薄弱学校改造工程375个。扩大异地就医即时结算范围，实现与西安等市外22个定点医疗机构联网结算。新建、改造6个标准化剧场，十一届中国艺术节榆林分会场精彩纷呈，《丝路欢歌》荣获全国“群星奖”，“榆林古城六楼民俗文化展演”入选国家公共文化服务体系示范项目。启动十四届全运会分会场筹备工作，圆满完成省七届少儿运动会承办任务。大力推进“大众创业、万众创新”，发放创业贷款5.15亿元，新增城镇就业2.92万人。稳步提高社保水平，在全省率先启动新农合市级统筹，实现城乡居民养老保险全覆盖，城乡低保标准分别提高到每人每年6000元和3015元。深入推进“平安榆林”建设，深刻汲取“1·06”刘家峁煤矿、“10·24”新民爆炸教训，从严开展易爆物品、社会治理难点大排查和安全生产大整顿等专项行动，着力化解矛盾纠纷和信访积案，社会大局总体稳定。

过去一年，我们着力加强政府自身建设，深入开展“两学一做”学习教育，党员干部“四个意识”进一步增强；完成市县两级政务服务中心标准化建设，政务服务水平大幅提升；不断强化行政监察和审计监督力度，“三公经费”下降8%；自觉接受人大依法监督、政协民主监督和社会舆论监督，人大代表建议、政协委员提案办复率均达100%；严格落实全面从严治党主体责任，驰而不息纠正“四风”，严厉整治不作为、慢作为和乱作为，政府执行力、公信力和服务水平不断提升。

此外，我们在质监、统计、司法、物价、人防、供销、气象、地震、新闻广电、扶残助残、民族宗教、国防和军队建设等方面都取得了新成绩。

各位代表，在困难多、任务重、挑战大的2016年，我们沉着应对、合力攻坚，稳住了经济、提升了信心，取得了来之不易的发展成绩。这是省委、省政府坚强领导、关怀支持的结果，是市委总揽全局、科学决策的结果，是全市广大干部群众同心同德、奋力拼搏的结果。特别是在座的同志们，肩负了比以往更沉重的压力，经受了比以往更严峻的考验，付出了比以往更艰辛的努力。在此，我谨代表榆林市人民政府，向全市人民，向各位人大代表、政协委员，向积极参与榆林经济建设的中省驻榆企业和国内外客商，向关心支持榆林发展的社会各界人士，向驻榆部队指战员、武警官兵和公安干警表示衷心的感谢，并致以崇高的敬意！

在看到成绩的同时，我们也清醒地认识到，当前我市发展中还面临一些突出的问题和挑战，主要表现在：产业结构单一，民间投资不活跃，经济企稳回升的基础还不牢固；营商环境不优，科技、人才等要素支撑不足，摆脱资源依赖、加快转型升级的任务仍很艰巨；城乡、区域发展仍不平衡，镇村基础设施及整体面貌还比较落后；安全生产、生态环保、社会治理等领域的风险不容忽视；公共服务供给不足，群众关心的就业、医疗、养老等工作仍需加强；特别是一些干部群众和企业家的思想解放不够，市场化意识不强，内陆经济的思维定势亟待跳出。对此，我们要高度重视，认真对待，采取有力举措加以解决。

二、2017年工作安排

今年是党的十九大召开之年，也是加快追赶超越的关键一年。当前，世界经济深度调整，我国经济稳健运行，陕西新一轮大发展的序幕已经拉开，榆林经济筑底回升、企稳向好的态势日趋明显。市第四次党代会描绘了全面建成更高质量小康榆林、阔步迈进全国地级市综合实力50强的宏伟蓝图。我们一定要抢抓国家深化供给侧结构性改革的重大机遇，抢占陕西追赶超越“天时、地利、人和”的发展先机，珍惜榆林心齐、气顺、劲足的发展局面，以改革创新的精神和一往无前的勇气，自加压力、主动作为，奋力谱写榆林经济社会转型升级、追赶超越的发展新篇章！

今年政府工作总体要求是：全面贯彻党的十八大和十八届三中、四中、五中、六中全会精神，深入学习习近平总书记系列重要讲话和来陕视察重要讲话精神，统筹推进“五位一体”总体布局和协调推进“四个全面”战略布局，全面落实“追赶超越”和“五个扎实”要求，聚焦“三区一高地”战略定位和“两个率先”目标，以提高发展质量和效益为中心，以推进供给侧结构性改革为主线，以落实“三项机制”为保障，坚持稳中求进总基调，自觉践行发展新理念，统筹做好稳增长、促改革、调结构、惠民生、防风险各项工作，坚决守住安全生产、生态环境、社会稳定、党风廉政四条底线，全力促进经济平稳健康发展和社会和谐稳定，以优异成绩迎接党的十九大和省第十三次党代会胜利召开。

主要预期目标是：地区生产总值增长6.5%以上，全社会固定资产投资增长5%，地方财政收入增长3.6%，社会消费品零售总额增长5%，城乡居民收入分别增长7%和8%；各项工作与标杆地市差距明显缩小，追赶超越取得显著进展。

围绕总体要求和预期目标，重点做好八方面工作：

（一）统筹推进稳增长调结构，巩固经济企稳向好势头。

全力振兴实体经济。突出分类指导、分业施策，强化经济运行考核调度等常态化机制，全力支持企业释放产能、扩大销售，确保规上工业增加值增长4%以上。全面清理规范涉企收费，用足用好中省减税降费让利政策措施，切实降低企业用工、用地、用能等经营性和制度性交易成本。实施质量强市和品牌引领战略，推进国家煤及盐化工产品检验中心建设，开展“增品种、创品牌、提品质”行动，持续提升榆林好产品市场竞争力。支持中省驻榆国有企业发展壮大，鼓励与中小企业开展协作配套，发挥好大企业、大集团的支撑引领作用。毫不动摇地支持民营企业家干事创业，深入实施中小企业提升行动计划和“抓大育小”工程，引导民营企业完善现代企业制度，用好中小企业发展资金和“助保贷”，支持开展小微企业票据贴现业务，继续选聘优秀高校毕业生到民营企业工作，加快个转企、企转规步伐，全年新增规上工业企业30户、限上批零住餐企业75户、规上服务业企业70户。打好棚改货币化安置等去库存政策“组合拳”，推动房地产市场健康发展。鼓励住宿餐饮业品牌化经营，支持物美等商贸连锁企业向县区拓展，促进传统商业向“线上线下”“体验销售”等新模式转变；开展无车承运、多式联运试点，推进横山海荣物流、定边公铁联运等物流园区建设，全年服务业比重提高到35%以上。

扎实推进能化基地高端化。坚持“三个转化”，按照“优煤、稳油、扩气、增电、强化工”的思路，积极构建高端化、多元化、集群化现代产业新体系。持续做好煤的文章，加快整合煤矿转产投运，推进煤炭产能减量置换，实行差别化去产能政策，支持神华、中煤、陕煤等企业释放先进产能，启动“榆林煤”中国驰名商标申报工作，全力扩大榆林煤终端市场份额。支持长庆、延长、中盐等中省企业稳产扩产，原油和天然气产量分别稳定在1000万吨以上和160亿方左右，盐及盐化工产量力争突破400万吨。加快榆横—潍坊、神木—河北南网等3条电力外送通道及4个配套电厂建设，力争陕北—湖北电力外送通道及5个配套电源点取得核准。启动天元回转热解、神木龙成提质煤、陕煤化国富炉等百万吨级粉煤干馏项目，加快兰炭生产清洁化改造和产品质量体系标准化建设，推进富油环烷基油等煤焦油精深加工项目，站稳煤炭热解产业发展制高点。抓好华电煤基芳烃、乌苏海则煤制天然气、延长靖边煤油气综合利用二期等转化项目，力争未来能源煤间接液化二期、兖州煤业聚甲氧基二甲醚、神华乙二醇、新疆天业乙二醇、清涧天然气制合成油等一批引领转型升级的重大项目落地，推动能化工业向下游高附加值产业链延伸。

大力发展文化旅游产业。成立市旅发委，实施全域旅游战略，高起点编制完成全市旅游发展总体规划以及乡村旅游、红色旅游等专项规划，促进文化、旅游与相关产业互动融合，加快把文化旅游业打造成新的支柱产业。大力发展文化创意、节庆会展、文艺演出等产业，抓好文化企业、人才的引进、培育工作，文化产业增加值增长15%以上。支持白云山、红碱淖5A级景区创建，完成统万城遗址公园一期工程，开工建设石峁遗址公园，全力打造一批龙头景区。加快特色客栈、户外营地、景区道路等基础设施建设，推进旅游“厕所革命”，着力提升“吃住行游购娱”要素供给。瞄准现代旅游需求，包装推介长城边塞、黄河风情、转战陕北红色体验等精品旅游线路，扩大“榆林过大年”“清爽榆林”等系列活动影响力，举办沙漠主题、冰雪运动、民俗体验、摄影写生等各类活动，打造陕北印象集中体验地，全年游客接待量、旅游收入分别增长20%和33%。

培育壮大新兴产业。做好光伏领跑者计划报批和实施工作，破解光伏用地、上网等难题，加快小壕兔风电场、砖井光伏电站、靖边多能互补集成优化示范等项目建设，新增新能源装机80万千瓦以上。建成佳县多晶硅、绥德镁合金项目，推进陕西有色新材料二期工程，加快煤基石蜡、陶瓷膜、碳纤维等项目落地，打造具有全球竞争力的新材料基地。开工建设靖边无人机测试中心、空港鹤翼无人机制造等项目，加快推进蓝时陕汽电动汽车及电池、定边正信光伏组件、中核集团风机塔筒等项目落地。紧盯未来产业发展方向，引进布局大数据、物联网、石墨烯、智能制造、3D打印等高新技术产业。深化与京东、阿里巴巴、中国网库等互联网企业合作，支持定边国泰、米脂淘米等本地电商做大做强，推动信息经济和分享经济突破发展。

（二）深入实施项目带动战略，不断增强追赶超越后劲。

持续扩大有效投资。始终把项目建设作为推动经济发展的主抓手，开展“重点项目建设年”活动，全面强化项目的策划、组织和实施，在全市范围内掀起一轮项目建设热潮。扎实推进200个市级重点项目建设，强化对县级重点项目的督导考核，确保西湾露天煤矿、榆神热电厂等35个项目建成投运，争取小保当一号煤矿、大海则煤矿、中煤煤制烯烃二期等重大项目核准。加强项目包装策划工作，瞄准重点领域、重点产业、重点地区，全年入库重点项目150个。全力推进政府与社会资本合作（PPP）项目，重点围绕公共服务领域推介PPP示范项目100个，年内新落地建设10个以上。用好用活国家支持西部基础设施建设政策，加大各类专项债券和政策性贷款争取力度，努力在交通、电力、水利、棚改等领域谋划实施一批重大项目，全力稳住投资增长态势。

扎实开展精准招商。坚持“市场换产业、资源换项目、项目换投资”的理念，创新招商引资政策，建立大招商工作机制，把招商引资作为衡量干部工作业绩和地方发展环境的重要标尺，夯实各级各部门招商责任。全面开展精准招商，围绕优势产业、骨干企业和产业链关键节点，采取智库招商、以商招商、产业链招商等多种方式，着力引进一批关联度高、带动力强的龙头型、科技型、配套型项目。抓住东部沿海产业转移、北京非首都功能疏解和与江苏对口扶贫等机遇，推动“央企进陕”项目落地，力争在引进填补产业空白、延长产业链条、资源循环利用等项目上取得突破。切实提高招商引资合同履约率、资金到位率和项目开工率，确保招商引资到位资金达到660亿元。

不断提高对外开放水平。加快融入陕西自贸区和“一带一路”开放大格局，实施外贸企业培育工程，继续加强通关便利化建设，加快资本、产能、技术等要素引进来、走出去步伐，拓展“海外陕西”的榆林板块。加强与京津冀、长三角、珠三角等地区的联系对接，在能源供应、招才引智、文化旅游等方面开展全方位合作。抢抓中蒙俄经济走廊建设机遇，打开俄罗斯、蒙古等上合组织国家化工、农副产品新市场，打造陕西向北开放新高地。深化呼包银榆经济区、陕甘宁革命老区、陕甘宁蒙晋沿黄地区区域合作，继续推进基础设施互联互通和经贸文化互动。加强与西安、太原、郑州等地经贸平台的交流协作，提升榆林“煤博会”办会水平，打造国家级、国际性的经贸会展平台。

铁腕整治投资环境。开展投资环境专项整治行动，化解村企纠纷，打击非法阻工，惩戒推诿扯皮，根治吃拿卡要，以零容忍态度整改百姓反映集中、企业反映强烈、社会反映普遍的突出问题，迅速改善榆林投资环境。建立市、县（园区）、乡镇三级投资环境责任体系，开展投资环境考核和排名公示，强化“投资环境110”职能，对不作为、慢作为、乱作为逐级问责，为企业发展、项目建设保驾护航。清理规范中介服务机构，加强招投标管理，强化重点项目周边、运煤通道沿线治安环境整治，严厉打击村霸等农村恶势力，营造重信守诺、尊商富商的投资新环境。

（三）扎实推进农业供给侧结构性改革，促进农业提质增效农民持续增收。

加快现代特色农业基地建设。按照“稳粮、强畜、扩果、优菜”工作思路，集中力量调优农业产业结构，促进农业增产增值增效。继续调减非优势产区玉米种植规模，扩大杂粮、油料、中药材等特色农作物种植面积，实施马铃薯良种繁供“一亩田”、谷子良种统繁统供、水肥一体化等重点工程，粮食总产量稳定在145万吨左右。支持蔬菜、时令水果等设施农业发展，壮大山地苹果、核桃、大扁杏、海红果等特色林果业，新建优质山地苹果6万亩，新增核桃等其他经济林6万亩，改造红枣低产园5万亩；积极推动畜牧业提质增效，推进肉羊适度规模标准化养殖。培育新型经营主体，新增专业合作社100个、家庭农场150个，培养职业农民2000人。加强现代农业园区建设，发挥好榆林农业科技示范园区的辐射带动作用，新认定市级以上农业园区10个。提高农业生产保障能力，加强高标准农田建设，推广节水灌溉农田5.2万亩；健全农业科技服务体系，支持农科院产学研一体化建设，抓好科技特派员和专家大院工作。健全农产品质量安全追溯体系，新认证“三品一标”农产品35个，抓好传统名优品牌的保护、提升和整合工作，推广榆林优质农产品标识，加大市场推介和开拓力度，让更多榆林绿色农产品走进全国百姓厨房。

促进一二三产业融合发展。实施农村产业融合发展示范工程，支持榆阳区开展国家农村一二三产业融合发展试点，提升10个市级休闲农业示范点，新打造一批集特色农业、农事体验、乡村旅游于一体的多业态复合型田园综合体。下工夫抓好农产品加工业发展，培育“农产品加工明星企业”，推进预冷、保鲜贮藏等设施建设，发展黄芪等中药材精深加工，加快马铃薯主食化产品开发，建成定边10万吨全粉、薯条生产线，新建2家10万只以上肉羊屠宰加工厂，农产品加工转化率达到35%以上。促进物联网技术向农业生产、流通等领域渗透延伸，支持7个电子商务进农村综合示范县（区）建设，新建5个市级农产品电商孵化基地、10家市级农产品电商示范企业。

深化农村综合改革。以放活农村土地经营权为突破口，深入推进“三权分置”改革，完成农村土地确权登记颁证尾留工作，支持发展土地流转、股份合作、代耕代种、土地托管等多种形式的适度规模经营。稳步开展农民住房财产权和土地经营权抵押贷款、政策性农业保险试点，创新完善林权抵押贷款机制，拓宽“三农”融资渠道，唤醒“沉睡的土地资本”。积极推进农村集体产权制度改革，开展榆阳、靖边、子洲农村集体资产清产核资试点，建立完善集体资产股权登记制度，加快县区农村产权交易中心建设，推动农村资源变资产、资金变股金、农民变股东。

（四）坚持以人为核心的新型城镇化，推进城乡区域协调发展。

全面启动“两廊一带”建设。高标准规划、高规格推动长城沿线新型工业走廊、无定河生态廊道和沿黄生态经济带建设，重构市域空间开发格局，更大力度更高视野统筹城乡区域协调发展。支持神木、靖边撤县设市，加强产业协作配套，推动煤油气等能化产业集群化发展，进一步引导人口、产业向长城沿线集聚。启动无定河全线综合整治，坚持净水、治水并举，实施绥德、横山等5县区防洪工程，建成杨桥畔镇、镇川镇2个污水处理厂，全部取缔排污不达标企业，基本消除县城黑臭水体。完成沿黄公路三期工程，建设沿黄绿色走廊，积极配合黄河国家公园申报工作，支持绥德建设沿黄区域中心城市，着力打好生态、旅游两张牌，推动沿黄地区由“穷腰带”向“富腰带”转变。

打造区域中心城市。坚持规划立市，加快完善城市功能，持续提升榆林在陕甘宁蒙晋接壤区域的影响力和辐射带动能力。高质量完成第五版城市总体规划修编工作，严格规划执行管控，维护规划的权威性、严肃性。加快推进迎宾大道西段、怀德路东延等22条道路改造工程，实施驼城家园等10个棚改项目；启动绿道慢行系统建设，优化公交线路和站点布局，多措并举化解停车难问题；推进西南新区、空港生态区与主城区基础设施互联互通。完成文化艺术中心主体工程，启动图书馆、博物馆、群艺馆、规划展览馆和会展中心、体育中心建设，打造城市“会客厅”；实施榆溪河生态长廊二期工程，因地制宜建设小游园、小绿地、小广场，提升城市品位。加快万达广场项目落地，打造高新区高品质商圈，加快老城区和西沙、东沙片区商业设施升级改造，优化消费环境，聚集人气商气。加快发展研发设计、商务咨询、融资租赁、物流配送、教育培训等生产性服务业，全面提升城市综合服务功能。围绕“一巩固六创建”，推进城市精细化管理，做好迎接国家卫生城市复审工作，加强建筑风貌管控，支持学校、机关单位体育设施向社会开放，开展占道经营、门脸牌匾等市容市貌专项整治行动，从细从小改善城市面貌，努力建设让群众更有自豪感、幸福感、归属感的美好家园。

加快新型城镇化建设。支持县区统筹推进水电路气暖、垃圾污水处理等基础设施和美化亮化绿化等公共服务项目，加快实现集中供水、供热和垃圾污水达标处理，提升县城宜居宜业水平和人口吸纳能力。启动“一县一镇”示范工程，集中力量提升重点镇公共服务供给能力，支持有条件的重点镇实施集中供热和气化工程；抓好波罗、名州、佳芦3个省级文化旅游名镇和大保当、四十里铺2个市级重点镇建设，精心打造一批特色小镇，促进“产、城、人、文”融合，推动新型城镇化内涵式发展。全力推进美丽乡村建设，启动新一轮城乡环境卫生综合整治行动，实施农村安全饮水工程420处，新建农村公路1000公里，建制村全部通水泥（沥青）路，改善农村人居环境，创建120个美丽乡村；加强传统村落保护发掘，推动民宿产业与特色农业、乡村旅游交融发展，让美丽乡村成为全域旅游战略的重要支撑。

提升县域发展水平。积极推进北部县区转型发展、南部六县绿色发展、各类园区创新发展，不断增强区域发展协调性。深入推进神府民营经济转型升级试验区建设，持续优化北部县区产业结构，加快摆脱“一煤独大”“油主沉浮”的局面，推动北部县区走在全省县域经济发展前列。持续加大振南力度，完善“四位一体”工作机制，实施和布局一批骨干项目，研究推广“飞地经济”，切实增强南部县自我发展能力。创新园区建设和运营模式，推动榆神工业区、榆横工业区打造世界一流煤化工产业和高新技术产业集聚区；进一步明晰县级产业园区发展定位、功能布局，推进镇园一体、产城融合；持续加大基础设施投入，建成运营9个污水处理厂、11个垃圾处理场，提升园区承载能力，打造县域经济发展主引擎。

（五）全力以赴破难题补短板，持续提升发展保障能力。

狠抓基础设施建设。扎实推进准朔、蒙华和靖神铁路（榆林段）建设，力争延安—榆林高铁年内开工。加快吴定、绥延高速建设，继续推进神佳米高速，确保G307吴堡至绥德、G210刘官寨至横山城区等7条公路建成通车。启动榆林榆阳机场二期扩建，加快神木通用机场、府谷和定边民用机场建设。抓好定边扬黄改建提升工程，建成清涧县城引黄、榆佳工业园区供水工程，加快绥德、米脂、佳县、子洲等县城后备水源地建设，做好大泉引水等重大水利项目前期工作。全面启动“智慧榆林”建设，加快推进光网入户城乡全覆盖和4G网络行政村全覆盖。

加强财税金融工作。做好“盘活、撬动”两篇文章，充分发挥财政资金杠杆作用，增强服务经济社会发展和保障民生的能力。加大国有资产和存量资金盘活整合力度，发挥好国有资产的融资融券功能。加快设立不低于200亿元的城市基础设施、产业转型、文化旅游等产业基金，引导和撬动社会资本。综合运用财政贴息、融资担保、以奖代补、政府购买服务等方式支持项目建设，发挥好财政资金的引导带动作用。争取新一轮政府存量债务置换指标，支持县区发行一批新的地方政府债券。创新金融产品和服务，完善政府、银行、担保和保险机构分担企业信贷风险合作机制，大力发展融资租赁、信托管理等金融新业态，引导更多金融活水流入实体经济。继续做好企业上市培育工作，重点支持北元化工在主板上市融资。深化与省金控、金资战略合作，参与组建第二家省金融资产管理公司。强化政银企对接，通过贷款重组、“债转股”等方式降低不良贷款规模，加大类金融机构监管力度，保持打击非法集资高压态势，守住不发生区域性系统性金融风险底线。

强化科技创新支撑。启动国家创新型城市建设，实施科技型企业“333”行动，加快培育高新技术企业，科技进步贡献率达到40%以上。促进“政产学研用”结合，统筹中省市科技资源，用好科技成果引导转化基金，支持榆林煤化工技术研发中心、新材料工程中心以及“星创天地”、众创空间等创新载体建设，开展煤炭分质利用、镁合金冶炼与加工、旱作农业节水、小杂粮新品种选育、陕北白绒山羊新品种选育等关键技术研究，推动更多工程技术、科研成果在榆转化。依法保护知识产权和创新收益，发挥好企业家在把握创新方向、凝聚创新人才、筹措创新投入等方面的重要作用。深入实施人才优先发展战略，进一步完善人才引进、培养、评价、激励机制，营造拴心留人的人才环境；大力实施“522”人才计划，抓好第四批刚性引进人才工作，开展“十大类人才精准培训工程”；建立创新创业和科技成果转化激励机制，激发各类人才的创新创造活力。

（六）加快推进关键性改革，更好发挥改革的先导和牵引作用。

纵深推进“放管服”改革。严格执行权力清单制度，再取消、下放、调整一批“含金量”高的审批事项。深化“多证合一、一照一证”和“证照分离”改革，持续推进企业登记注册简易化和便利化。启动投资项目全流程在线并联审批制度改革，推动“多规合一”各项工作走在全国前列。全面推行“双随机一公开”监管，提升监管的公平性、规范性和有效性。推进“互联网+政务服务”，公布市县乡三级公共服务事项清单，开展市政务服务中心全国社会管理和公共服务综合标准化试点，建成市县政务中心网上审批大厅。加快组建公共资源交易中心，确保上半年投入运行。

加强重点领域改革。推动市属国企统一监管，深化市属国企“一企一策”改革，加快榆能、城投等国企整合重组，完善法人治理结构。落实国有林场改革要求，创新完善国有林场森林管护、造林营林等机制。有序推进事业单位分类改革，继续做好中职教育资源整合。深化电力体制改革，扩大电力直接交易，推进电力多边交易，探索无限价挂牌交易，全力打造低电价区。深化公立医院综合改革，推动分级诊疗、“两保合一”、药品采购、医保支付、综合监管等五项制度落实。加快城市执法体制改革，着力提升城市管理效能。继续完善不动产统一登记制度，实现规范登记、信息共享。

（七）系统推进生态文明建设，守护好榆林的蓝天绿水青山。

重拳治理环境污染。坚决向环境污染“亮剑”，不要污染的GDP。全面实行河长制，夯实市、县、乡三级河长责任。抓好中心城区“四河”治理，堵住榆溪河20个污水直排口，开工建设市第二、第三污水处理厂，全面消除中心城区黑臭水体。关停排放不达标的火电机组，完成30万千瓦以上燃煤机组超低排放改造任务；加快煤改电、煤改气步伐，推广兰炭替换民用散煤。全面落实土壤污染防治行动计划，实施化肥、农药零增长行动，有效控制农业面源污染。实施农村生态文明建设示范工程，划定畜禽禁养区，推进畜禽养殖废弃物处理和资源化利用，农村生活垃圾无害化处理率达到75%。以“钉钉子”精神完成环保督查和自查问题整改，以零容忍态度打击各类环境违法行为。

持续优化生态环境。坚持生态优先战略，统筹推进山水林田湖一体化治理，加快建设国家生态保护与建设示范区。坚持柔性治水，完成榆林水系规划，推进煤矿疏干水综合利用，加大红碱淖等湿地保护力度。启动国家森林城市创建工作，继续实施林业十大工程，建设五个百万亩基地，新增营造林面积60万亩。实施一批国家水土保持重点工程，加固维修淤地坝850座，启动黄河粗泥沙集中来源区拦沙工程，治理水土流失面积1100平方公里。抓好采煤沉陷区治理、“三废”利用等生态修复工程。开展全民节能行动计划，强化重点用能单位节能管理。严格执行领导干部自然资源资产离任审计制度，落实生态环境损害责任终身追究制。

（八）脱贫攻坚保障民生，让人民群众有更多获得感。

决战决胜脱贫攻坚。认真落实“六个精准”和“五个一批”要求，加大各类扶贫资金整合力度，因地因户因人施策，确保全面率先脱贫不让一个人掉队。抓好脱贫、避灾、生态等移民搬迁工作，统筹推进产业扶贫、旅游扶贫、电商扶贫、光伏扶贫，提高贫困群众财产性和工资性收入。着力处理好“扶贫和扶志”“输血和造血”的关系，倡导良好村风家风，提升贫困群众自我发展能力。强化驻村帮扶、对口帮扶等包扶结对机制，深化与扬州市的对口扶贫协作，加快设立扶贫基金，健全脱贫长效机制，巩固脱贫攻坚成果。开展“脱贫攻坚质量年”活动，严格执行脱贫攻坚考核和第三方评估，杜绝数字脱贫、虚假脱贫，保质保量实现2.43万贫困人口脱贫、152个贫困村退出、7个贫困县区全部摘帽。

统筹发展社会事业。办好人民满意教育，完成学前教育二期行动计划，确保府谷、横山、吴堡、清涧、子洲成功创建国家义务教育均衡县（区），横山、吴堡、子洲通过“双高双普”省级评估认定；加强校长和师资队伍建设，着力提升高中办学质量；支持职业技术学院打造特色专业，扩大招生规模；支持榆林学院改善办学条件，提升综合竞争力。推进健康榆林建设，提升计划生育、重点专科和中医中药服务水平，推广医疗联合体和县乡村医疗服务一体化模式，促进优质医疗资源下沉，让百姓“好就医”“就好医”；加强公共卫生和重大疾病防控，加快市传染病医院、市精神卫生中心落地建设，建成医废处置二期项目；促进全民健康与全民健身融合，启动空港体育产业示范区等项目建设，加快补齐县城全民健身场地不足短板；举办各类全民健身体育赛事，提升榆林马拉松赛事规格，营造全民运动的良好氛围。健全公共文化服务体系，倡导全民阅读，强化公共场所免费书屋、阅读长廊等文化设施建设，提高万人公共文化设施面积；开展文明交通、文明餐桌等“十大文明行动”，提升城市文明形象和全民素质；承办好第八届全省艺术节，全面提升榆林文化影响力。

持续提升社保水平。切实加强就业工作，深入实施青年创业、大学生创业引领和农民工返乡创业“三大行动计划”，及时动态消除城镇零就业家庭，全年新增城镇就业2.85万人。积极推进全民参保计划，完成城乡居民基本医疗保险制度整合，完善大病保险制度，稳步提高合规医疗费用实际支付比例。扎实推进“医养结合”“智慧养老”等新型养老试点，组织建设一批老年人日间照料中心、托老所等养老服务机构，加快构建以居家为基础、社区为依托、机构为补充的多层次社会养老服务体系。做好农村留守儿童、留守妇女、留守老人的关爱服务工作，实现6岁以下残疾儿童康复救助全覆盖。

加强和创新社会治理。深化安全生产领域改革，加快国家危险化学品应急救援榆林基地和智慧安监建设，严厉打击煤矿无证开采和超层越界开采等违法违规行为，突出抓好民爆物品安全管理，坚决遏制重特大安全生产事故发生。强化食品药品监管，保障群众“舌尖上的安全”。完善城乡社区服务和管理标准，加强社会工作人才和社区队伍建设，实现政府治理和社会调解、居民自治良性互动。推进城市网格化管理，完善信息化、立体化社会治安防控、公共安全防控和互联网动态管控体系。加强信用体系建设，建立企业和个人征信数据库，严厉打击金融欺诈、恶意逃废债等违法行为，打造“诚信榆林”。有效化解信访积案，依法打击非访、缠访和闹访。强化网络舆情监测和应急处突，加大对吸毒贩毒、流窜作案、“两抢一盗”、电信诈骗、涉众型经济犯罪的防范打击力度，切实保障人民群众生命财产安全。

三、切实加强政府自身建设

追赶超越的使命艰巨而繁重，群众对美好生活的期待现实而热切。我们将以时不我待的紧迫感、舍我其谁的使命感和功成不必在我的责任感，努力建设政治坚定、依法行政、运转高效、为民务实、清正廉洁的人民满意政府。

始终做到立场坚定、心有方向。深入学习领会习近平总书记治国理政的新理念、新思想、新战略，进一步增强“四个意识”特别是核心意识、看齐意识，始终在思想上、政治上、行动上与以习近平同志为核心的党中央保持高度一致，自觉向党中央看齐，向党的理论和路线方针政策看齐。自觉践行以人民为中心的发展思想，聚焦追赶超越中心任务和“五个扎实”要求，确保中省重大部署和市委各项决策落地生根。

始终做到崇尚法治、履职尽责。扎实开展“法治政府建设年”活动，落实法治政府建设实施纲要，健全政府法律顾问制度，严格执行重大行政决策法定程序，全面提升依法行政能力和水平。落实公众参与、专家论证、风险评估、合法性审查和集体决策机制，加强智库建设，全面提升政府决策的科学性。自觉接受人大法律监督、政协民主监督和社会监督、舆论监督，加强与各民主党派、工商联、无党派人士的沟通联系。严格落实重大事项报告制度，全面提高人大代表议案建议和政协委员提案的办理质量。深入开展“七五”普法工作，营造全社会尊法学法、守法用法的良好社会氛围。

始终做到敢于担当、奋发有为。强化“三项机制”保障作用，为担当者担当、为负责者负责、为改革创新者撑腰鼓劲，让广大干部敢干事、能干事、干成事。全面治理为官不为、为官乱为和懒政怠政行为，倒逼各级干部在其位、谋其政，敢担当、善作为。建立健全政府公职人员培训、调研等各项学习制度，主动适应追赶超越新要求。围绕追赶超越“任务书”“路线图”，严格执行月度排名、季度点评、年度考核制度，实行工作任务菜单式分解、责任清单式管理，让追赶超越成为各级政府的自觉行动。

始终做到高效服务、立说立行。全面整治“门好进、脸好看、话好听、事不办”的行为，深入开展“减证便民”专项行动，大兴“马上就办”之风，让群众办事更方便、少跑路、不堵心。坚持“问需于民、问政于民、问计于民”，走好群众路线，办好市长信箱、政府门户网站，畅通社情民意表达渠道，确保群众合理诉求早发现、早回应、早解决。着力提升政务公开的力度，用好官方微博、微信等各类新媒体平台，做好重大政策、权威消息的发布和解读，讲好“榆林故事”，传播好“榆林声音”。

始终做到严守规矩、廉洁奉公。严格落实全面从严治党要求，切实履行“一岗双责”，认真执行《准则》和《条例》，始终把纪律和规矩挺在前面，严肃党内政治生活。加强对政府采购、工程招投标、公共资源交易等重点领域监管，前移审计、监察监督关口，扎紧制度的笼子，确保资金安全、项目安全。严格落实八项规定精神，压缩财政一般经费支出，用政府的紧日子保障群众的好日子。紧盯侵犯群众切身利益的“微腐败”，无禁区、全覆盖、零容忍惩治腐败，营造风清气正的发展环境。

各位代表、同志们，唯其艰难，方显勇毅；唯其磨砺，始得玉成。榆林正处在逆水行舟、滚石上山的关键时期，加快追赶超越已经成为当前最大的任务。让我们更加紧密地团结在以习近平同志为核心的党中央周围，在省委、省政府和市委的坚强领导下，撸起袖子加油干，努力在全省追赶超越中实现好“榆林使命”，为全面建成更高质量的小康榆林而努力奋斗！
